# Sodium Alginate/Carboxymethyl Chitosan Hydrogel Microbeads for Antibiotic Adsorption in Single and Binary Systems

**DOI:** 10.3390/gels11080646

**Published:** 2025-08-14

**Authors:** Zhisong Qian, Xinpeng Li, Gege Yan, Xiaoyong Chen, Mohd Shaiful Sajab, Gongtao Ding, Wan Nazihah Liyana Wan Jusoh

**Affiliations:** 1 Key Laboratory of Biotechnology and Bioengineering of State Ethnic Affairs Commission, Biomedical Research Center, Northwest Minzu University, Lanzhou 730030, China; zhisongqian797@gmail.com (Z.Q.); lixinpeng1234@gmail.com (X.L.); gegeyan225018@gmail.com (G.Y.); xiaoyongchen678@gmail.com (X.C.); 2 College of Life Science & Engineering, Northwest Minzu Northwest Minzu University, Lanzhou 730030, China; 3 College of Chemical Engineering, Northwest Minzu University, Lanzhou 730030, China; 4 Department of Chemical and Process Engineering, Faculty of Engineering and Built Environment, Universiti Kebangsaan Malaysia, Bangi 43600, Selangor, Malaysia; mohdshaiful@ukm.edu.my; 5 Research Center for Sustainable Process Technology (CESPRO), Faculty of Engineering and Built Environment, Universiti Kebangsaan Malaysia, Bangi 43600, Selangor, Malaysia

**Keywords:** adsorption, antibiotics, biomaterial, electrostatic spraying, hydrogel microbeads

## Abstract

The use of pharmaceuticals to treat human and animal diseases has resulted in the increase of antibiotic traces in the water system and soil, thus raising concerns about the environmental aspect. In this study, sodium alginate (SA) and carboxymethyl chitosan (CMCS) hydrogel microbeads were developed to enhance the adsorption of antibiotics by applying electrostatic spray in the fabrication of microbeads. Two hydrogel microbead sizes, SC-400 (~400 µm) and SC-2000 (~2000 µm), were used for the adsorption of tetracycline (TC) and ciprofloxacin (CIP) antibiotics in single and binary systems. The microbeads exhibited a good adsorption capacity and were able to achieve a maximum adsorption at pH 7 and 25 °C. Adsorption kinetics expressed suitability in the pseudo-second-order kinetic model for TC and CIP antibiotics. These results demonstrate that both single and binary systems align well with the Freundlich and Temkin isotherm models, indicating their suitability in explaining the adsorption mechanisms. These mechanisms predominantly involve electrostatic interactions between the SA/CMCS hydrogel microbeads and the antibiotics TC and CIP. This study highlights the capability of using SA/CMCS hydrogel microbeads for antibiotic removal and other environmental applications.

## 1. Introduction

Nowadays, antibiotics are widely used in medicine, agriculture, and veterinary fields which offer great advantages for disease control, prevention, and treatment. The increase in antibiotics usage has improved the advancement in healthcare services; however, it raises concerns regarding the long-term impact on both users and the ecosystem. Excessive and unregulated usage also leads to the accumulation of pharmaceutical compounds in the environment. Improper disposal and inadequate treatment of antibiotic waste have caused huge impacts, including the proliferation of antibiotic-resistant bacteria and the disruption of microbial communities. The interaction of antibiotics in different environments and systems also requires some optimization to enhance effectiveness. Subsequently, the development of innovative materials can help in controlling and managing the behavior of antibiotics in an effective manner.

Hydrogel, a soft matrix, has gained prominent attention due to its efficient absorbent ability, making it suitable for the application as a physisorbent and chemisorbent. It is three-dimensional and comprises crosslinked networks of hydrophilic polymers [1,2]. Hydrogel microbeads are extensively used as adsorbents for environmental remediation, biomedicine, agriculture, and the food industry. Hydrogel has high water retention capacity, controllable porosity, and biocompatibility, making it an exceptionally suitable biomaterial for antibiotic adsorption. It has been customized by various biomaterials, with a combination of sodium alginate (SA) and carboxymethyl chitosan (CMCS) being highly promising biomaterials. This is because the combined biomaterials possess good properties, such as biodegradability, biological attributes, high penetrability, and consistency [3,4].

Previous studies have investigated the application of SA/CMCS hydrogel in various fields. For instance, SA/CMCS with polycaprolactone (PCL) was prepared using the electrospinning technique and used in tissue engineering to test its physical properties and biocompatibility with MC3T3E1 osteoblast [5]. CMCS/SA hydrogel film is also used in the preservation of strawberries due to its ultrafast crosslinking using citric acid, low permeability of moisture, and expressed antibacterial properties [6]. The material was also incorporated with difenoconazole to explore pH responsive release for controlling wheat rot [7]. Additionally, several studies investigated the efficacy of hydrogel for adsorbing antibiotics, along with modifications using alginate and chitosan to improve the adsorptivity performance [8,9,10]. The findings denote that combining chitosan with graphene oxide resulted in a maximum of 67.6% ciprofloxacin removal, while the chitosan and biochar hydrogel beads were able to adsorb more than 64% of antibiotics even after the sixth regeneration.

Electrostatic spray technology was invented in the 1930s to improve the spray deposition on canopy, with the first application in 1940 [11]. Electrostatic spray deposition has been used in various fields such as the agricultural, food, coatings, biomedical, and manufacturing industries. Electrostatic spray refers to microfluidic spray in which the liquid sample forms droplets through the external force generated from the electrostatic field [12,13,14]. The droplets dispersion occurs in a Taylor cone shape, forms uniform size and shape, and can reach nanosize by controlling some parameters [12,15]. Typically, the electrostatic spray comprises four main components: a pump system, a nozzle, a high voltage power supply, and a metal collector. The liquid sample is pumped through the syringe and nozzle connected to the positive electrode, and the free charge at the tip of the cone shape increases the dispersion of droplets [12]. The strength of the electric field, liquid flow rate, and nozzle diameter are among the significant process parameters that can affect the fabrication of droplets [14]. Additionally, the electrostatic spray can be influenced by operation parameters (voltage, flow rate, distance), liquid properties (concentration, surface tension, conductivity), and environmental conditions (temperature, pressure), thus adding more influencing factors toward bead formation [12].

Following the benefits of SA/CMCS and the electrospray technique, this study aims to systematically explore the adsorption mechanism and behavior of hydrogel microbeads in antibiotic adsorption and removal. Although numerous studies have explored the application of SA/CMCS biomaterials, there remains a limited understanding of the effect of microbeads generated via electrostatic spraying on antibiotic adsorption. The dependency of antibiotic adsorption is emphasized based on size-dependent microbeads in both single and binary antibiotic systems. The study focuses on understanding how the size of microbeads influences adsorption kinetics, equilibrium capacity, and adsorption interaction. The findings shall contribute to effective solutions for controlling the contamination of antibiotics and provide practical recommendations for targeted environmental and health control.

## 2. Results and Discussion

### 2.1. Effect of pH on the Antibiotic Adsorption

**Figure 1:**
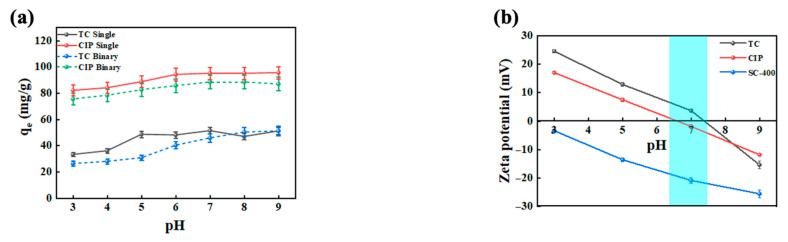
(**a**) Effect of pH (3.1–2.0) on the single and binary solutions and (**b**) zeta potential of pH (3.0–9.0) for microbeads (SC-400) and antibiotic solution.

The antibiotic adsorption by microbeads was analyzed at different pH and temperatures. Figure 1a illustrates the variation in removal efficiency presented by the amount of adsorbate adsorbed, *q*_e_ of CIP and TC by electrostatically sprayed SA/CMCS microbeads (SC-400) with changes in pH. As the pH increased from 3.0 to 9.0, the hydrogel microbeads demonstrated higher adsorption efficiency for single antibiotics compared to binary antibiotic solutions. For single CIP antibiotic solutions, the adsorption capacity (*q*_e_) initially increased and then slightly reduced before reaching the maximum adsorption of 95 ± 2% at pH 7.0. For single TC antibiotic solutions, the *q*_e_ increased, plateaued, and then decreased, with the highest adsorption of 51 ± 2% at pH 7.0. These results are supported by the TC and CIP removal using negatively charged copper nanoparticles, where the optimum condition at pH 7 gives the maximum removal ability [16,17].

This study observed that a stronger pH-dependent reaction occurred in TC adsorption compared to CIP. This was potentially due to the gradual deprotonation of -COOH groups into -COO^−^ under neutral conditions at pH 7 [18]. This deprotonation enhances the electrostatic interaction between positively charged TC and the negatively charged SA/CMCS hydrogel microbeads, thereby increasing the adsorption capacity of TC. This is possibly caused by the differences in the charged properties of the electrostatically sprayed SA/CMCS hydrogel microbeads and antibiotics at various pH levels. The enhanced adsorption may result from factors such as increased pore exposure or hydrogen bonding, rather than be purely affected by electrostatic interactions. Zeta potential analysis showed that the hydrogel microbeads remained negatively charged across all pH levels, with the magnitude of negativity increasing with pH, as shown in Figure 1b. The positively charged surface of TC and CIP also changed with pH. At pH values below 6.5–7.0, both TC and CIP carry a positive charge, which promotes electrostatic attraction to the negatively charged hydrogel microbeads. However, above this threshold, the charge inversion occurs and reduces the electrostatic interaction between antibiotics and microbeads. Thus, TC and CIP achieved an equilibrium adsorption capacity at pH 7.0, as shown by the graph in Figure 1a.

In binary solution systems, the adsorption trends of TC and CIP on the hydrogel microbeads with pH changes were similar to those in single systems. However, the adsorption amounts of TC and CIP were lower than single systems at the same pH. Notably, CIP adsorption decreased sharply and was significantly inhibited, indicating competitive adsorption between TC and CIP in binary systems. TC exhibited a higher adsorption priority as, CIP is less likely to carry a charge, hindering electrostatic interactions and reducing its adsorption efficiency.

### 2.2. Effect of Temperature on Antibiotic Adsorption

**Figure 2:**
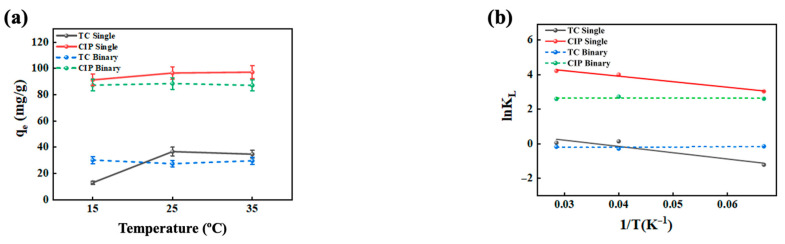
(**a**) Effect of temperature on the adsorption of single and binary systems of antibiotics; (**b**) the adsorption kinetics using Gibbs–Helmholtz.

As the solution temperature increased from 15 to 25 °C, the adsorption efficiency of TC and CIP on electrostatically sprayed SA/CMCS hydrogel microbeads gradually improved (see Figure 2a). Such increase was due to the enhanced mobility of antibiotics in the solution at higher temperatures, facilitating their contact with the hydrogel microbeads and subsequent adsorption. Additionally, the increase in temperature may expose more binding sites on the surface of the hydrogel microbeads, further enhancing adsorption efficiency [19]. However, the adsorption rate decreased when the temperature exceeded 25 °C. This was caused by the exothermic condition in the adsorption process of antibiotics on SA/CMCS hydrogel microbeads. The increase in thermal motion and temperature of the antibiotic solution weakened the electrostatic attraction between the antibiotics and the hydrogel microbeads [20,21]. The binary system of CIP expresses similar trends as a single system with lower adsorption efficiency. For the removal of TC binary system, it is higher at 15 °C and gradually becomes constant as the temperature increases. Meanwhile, for single TC, adsorption efficiency is lower compared to the binary system at lower temperature (15 °C) and significantly exhibits higher adsorption capacity as temperature increases. 

The adsorption results of TC and CIP at different temperatures were effectively modeled using the Gibbs–Helmholtz equation (Equations (1) and (2) in Section 4.3) and enthalpy and entropy (Equation (3)). The thermodynamic parameters are listed in Table 1 and Figure 2b. In both single and binary antibiotic systems, the Δ*G* values for TC adsorption on the hydrogel microbeads were consistently negative, indicating that the adsorption process is spontaneous and feasible, although with a lower adsorption amount. Conversely, the Δ*G* values for CIP adsorption were positive, suggesting that the process is non-spontaneous. The positive Δ*H* values for both antibiotics refer to the endothermic condition of the adsorption process, making higher temperatures favorable for adsorption. Additionally, the positive Δ*S* values for both antibiotics suggest an increase in disorder at the solid–liquid interface during adsorption. Notably, the ΔS values in the single antibiotic system were consistently higher than those in the binary system, indicating higher disorder occurrence in single system. The subsequent adsorption experiment in the next section was conducted at the optimal pH and temperature, pH 7 and 25 °C, respectively.

### 2.3. Adsorption Kinetics

**Figure 3:**
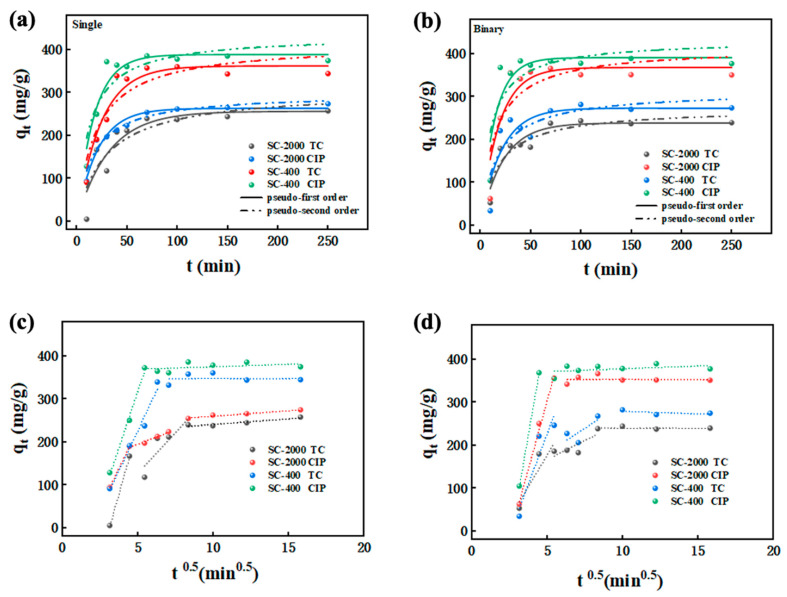
Adsorption kinetics models of pseudo-first and second order of (**a**) single system and (**b**) binary system; intraparticle diffusion model of (**c**) single system and (**d**) binary system.

The adsorption behavior of antibiotics was determined via the adsorption kinetics of antibiotics on electrostatically sprayed SA/CMCS hydrogel microbeads. The adsorption of single and binary systems was fitted using pseudo-first order and pseudo-second order kinetic models, with the equations (Equations (4) and (5)) expressed in Section 4.3. The calculated data based on the adsorption kinetics models is tabulated in Figure 3, and the fitting parameters are listed in Table 2.

In single and binary antibiotic solutions (Figure 3a,b), the adsorption of TC and CIP by the hydrogel microbeads initially entered a rapid adsorption phase, with a turning point occurring at approximately 50 min. It was followed by a slower adsorption phase until equilibrium was reached. For single system, the linear fit of the pseudo-first-order kinetic model showed a higher correlation coefficient (*R*^2^) compared to the pseudo-second-order model. This is aligned with the theoretical equilibrium adsorption *q*_e,_ values obtained from the pseudo-first-order model were closer to the experimental *q*_e_ values for all hydrogel samples. This indicates that the pseudo-first-order model provides better accuracy and consistency in describing the adsorption behavior and kinetics of CIP and TC in single antibiotic solutions.

For the binary system, the transition from rapid to slow adsorption was delayed to approximately 70 min. Additionally, in binary solutions, the adsorption of TC and CIP by the hydrogel microbeads showed better fitting results (*R*^2^) with the pseudo-second-order kinetic model, contrasting with the results from single antibiotic solutions. This suggests that adsorption in binary solutions is more complex, with interactions between the antibiotics influencing the adsorption behavior.

The study concludes that the adsorption process is physical and relatively straightforward, where the SA/CMCS hydrogel microbeads can interact directly with the antibiotics. A minor difference observed between single and binary adsorption resulted from competitive adsorption dynamic and the affinity of TC for active sites on the microbeads. This further implies a relatively fast adsorption rate, which is likely due to the electrostatic interactions between the anionic groups on the hydrogel microbeads and the cationic groups on the antibiotics, significantly influencing the overall adsorption rate [22,23]. It is also notable that the adsorption process affected by the size of microbeads. For example, the adsorption capacity of SC-2000 for TC recorded a 3% reduction from 262.9 mg·g^−1^ to 255.9 mg·g^−1^ compared to SC-400. The similar trend was observed for CIP, in which SC-400 offers higher adsorption capacity, confirming the effect of microbeads size.

To further understand the adsorption behavior of antibiotics, the intraparticle diffusion model was used to determine the role of diffusion in the adsorption, as described in Equation (6) in Section 4.3. As shown in Figure 3c,d, the adsorption of antibiotic TC by electrostatically sprayed SA/CMCS hydrogel microbeads fits well with the intraparticle diffusion model. This chemical adsorption process can be divided into three stages: (1) the transfer of antibiotic particles from the solution bulk to the outer surface of the adsorbent, (2) the transfer from the outer surface to the internal pores of the adsorbent, and (3) the adsorption equilibrium phase.

For the adsorption of CIP, the intraparticle diffusion model fits into two stages: (1) a rapid adsorption phase involving the transfer from the solution bulk to the particle surface and (2) an intraparticle or pore diffusion phase. This suggests that the migration rate of antibiotics from the bulk liquid to the surface of the hydrogel microbeads is relatively fast, while the internal diffusion rate is slower. The different fitting results can be attributed to the structural characteristics of TC molecules, which contain multiple aldehyde and ketone groups capable of forming hydrogen bonds with the adsorbent surface. These diverse interactions can further complicate the diffusion of TC within the particles, involving multiple adsorption and desorption processes, thus exhibiting three distinct stages in the intraparticle diffusion model.

### 2.4. Adsorption Isotherms

Beyond adsorption kinetics, adsorption isotherms were used to further elucidate the antibiotic adsorption process on electrostatically sprayed SA/CMCS hydrogel microbeads (SC-400 and SC-2000). The adsorption of antibiotics by the electrostatically sprayed SA/CMCS hydrogel microbeads was modeled using the linear equation of Langmuir, Freundlich, and Temkin isotherm models as expressed in Equations (7)–(9) in Section 4.3 and tabulated in Table 3.

**Figure 4:**
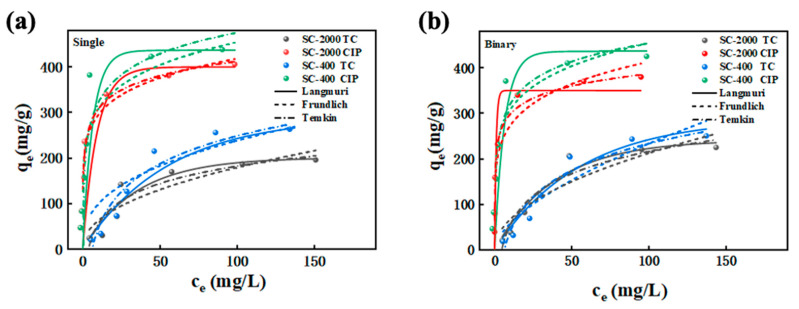
Adsorption isotherm for Langmuir, Freundlich, and Temkin models of (**a**) single system and (**b**) binary system.

The Langmuir isotherm model is suitable for monolayer adsorption on surfaces with identical and equivalent sites [24,25]. The adsorption isotherms of Langmuir, Freundlich, and Temkin are presented in Figure 4a,b for the single and binary systems, respectively. For the TC and CIP in both single and binary systems, the adsorption isotherms of antibiotics on SC-400 fitted the Langmuir model better, with *R*^2^ > 0.8517, indicating that the adsorption on SC-400 was primarily monolayer and uniform. On the other hand, the Freundlich model suggests multilayer adsorption on heterogeneous surfaces [24]. For the TC and CIP in single and binary systems, the adsorption isotherms of antibiotics on SC-2000 fitted the Freundlich model better, with *R*^2^ > 0.8349, indicating that adsorption on SC-2000 was predominantly multilayer and heterogeneous.

In single systems, the maximum adsorption capacity of for TC increased from 200.3 mg·g^−1^ (SC-2000) to 289.7 mg·g^−1^ (SC-400), indicating an improvement of 44.6%. For CIP, the maximum adsorption capacity increased from 399.5 mg·g^−1^ (SC-2000) to 436.1 mg·g^−1^ (SC-400), indicating an improvement of 9.2%. This shows that the size of hydrogel microbeads affects the adsorption capacity of antibiotics: the smaller size of the microbeads improved the adsorption capacity. In binary systems, the presence of both TC and CIP led to a slight reduction and increment in adsorption capacity compared to single systems. The adsorption capacity of SC-2000 for TC increased by 20.4%, while for CIP, it decreased by 12.4%, indicating competitive adsorption in the CIP binary system. Meanwhile, there is no changes for SC-400 in both the TC and CIP binary systems.

The Temkin model studies two parameter equation of adsorption isotherm on heterogenous solid surfaces with some assumption of uniform and infinite energy dispersion [26]. It is suitable for multilayer adsorption with uniform distribution and independent interactions among adsorption sites and one of the chemisorption systems [24]. Parameter *b* is negatively correlated with adsorption heat (Δ*H*), where a larger *b* value indicates lower adsorption heat and greater energy contribution from adsorption sites. SC-2000 had the highest *b* value for CIP adsorption, indicating the strongest adsorption effect and the highest adsorption capacity. The differing results can be attributed to the heterogeneous adsorption behavior of SA/CMCS hydrogel beads produced by the simple extrusion method. In contrast, the ES technology reduced the microbead size to 400 µm under high pressure, transforming the adsorption process into monolayer adsorption on the uniform surface of the hydrogel microbeads. This significantly enhanced the adsorption rate of SA/CMCS hydrogel microbeads for antibiotics.

### 2.5. Characterization and Adsorption Mechanisms

**Figure 5:**
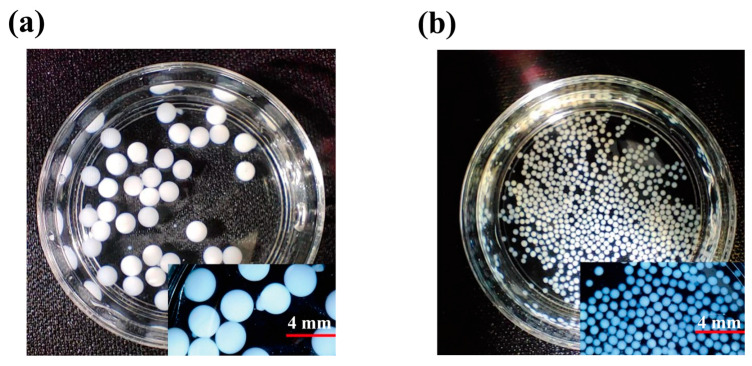
The image analysis of microbeads: (**a**) SC-2000; (**b**) SC-400.

**Figure 6:**
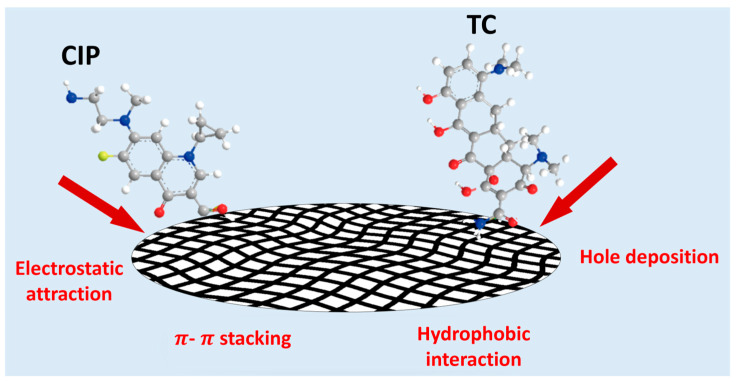
Illustration of adsorption mechanism for SA/CMCS hydrogel microbeads.

The microbead size for SC-400 is an average diameter of ~400 µm, whereas SC-2000 is ~2000 µm, and the microscopic images are shown in Figure 5. It is observed that the microbeads were able to retain their structural integrity after the adsorption experiment condition, and no significant disintegration of beads occurred. The adsorption mechanisms of antibiotics are influenced by various factors, including the structure, surface area, and chemical properties of the antibiotics, as well as the pore size, active sites, and interactions (π-π stacking, hydrophobic interactions, and electrostatic interactions) of the adsorbent [23,27,28]. Based on the adsorption studies discussed earlier, antibiotic adsorption using electrostatically sprayed SA/CMCS hydrogel microbeads under high pressure is physical adsorption driven by electrostatic interactions (see Figure 6).

**Figure 7:**
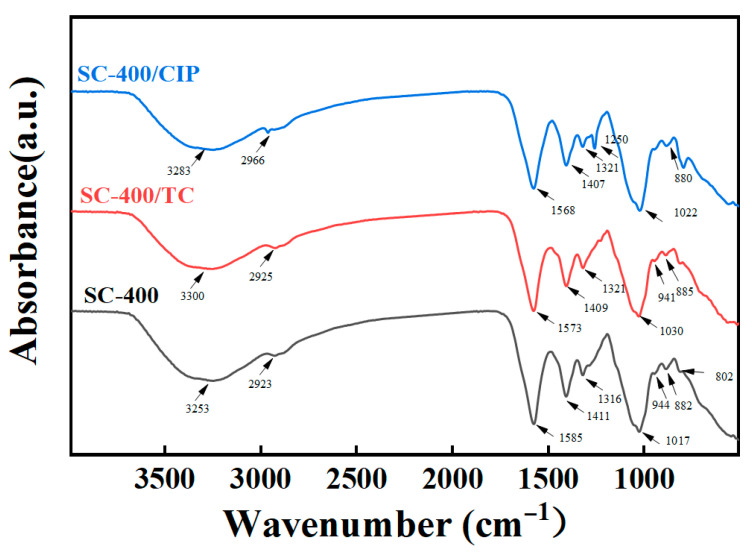
FTIR characterization of the SC-400 microbeads after antibiotic adsorption.

To elucidate the adsorption mechanism of antibiotics on SC-400, FTIR characterization was performed on the samples after antibiotic adsorption. The characterization (FTIR and ionic strength) focused on determining the effect on SC-400 as it showed significant adsorption performance. As shown in Figure 7, once TC or CIP was loaded onto SC-400, the adsorption peaks of the C-C double bond shifted from 1585 cm^−1^ to 1573 cm^−1^ and 1568 cm^−1^, respectively, which could be related to π-π interactions. It is well known that the structures of TC and CIP consist of aromatic ring frameworks, which can interact with the residual electrons on SC-400. For TC, cation-π bonding may be involved in the adsorption process due to the protonated amino groups located at the C4 ring of TC molecules and the π-electrons on SC-400. Furthermore, the characteristic absorption peak of SC-400 at 1017 cm^−1^, attributed to the C-O bond vibrations, shifted to 1030 cm^−1^ and 1022 cm^−1^ after TC and CIP adsorption, respectively. This provides further evidence of antibiotic adsorption on the SA/CMCS hydrogel microbeads.

**Figure 8:**
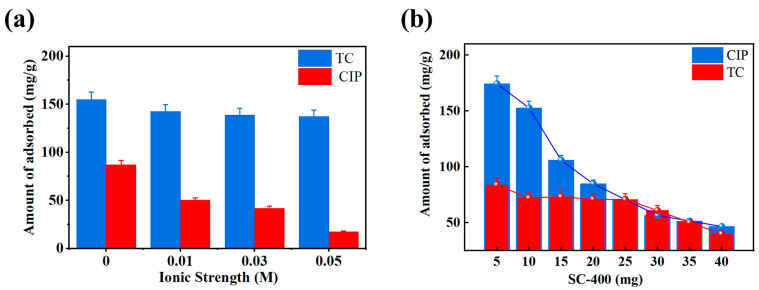
Adsorption performance of SC-400: (**a**) effect of ionic strength; (**b**) effect of amount of hydrogel microbeads in binary systems.

As shown in Figure 8a, ionic strength significantly affected the antibiotic adsorption capacity of SC-400. The results indicated that in the absence of NaCl (ionic strength = 0.00 M), SC-400 exhibited the highest adsorption capacities for TC and CIP, with values of 87.27 mg/g and 155.06 mg/g, respectively. When the ionic strength of NaCl increased to 0.05 M, the adsorption capacity for TC significantly decreased to 17.34 mg/g, which is 80% lower than the control group without NaCl. In contrast, the adsorption capacity for CIP was not significantly affected by ionic strength, indicating that electrostatic interactions play a key role in the adsorption of TC. The ionic strength also impacts the adsorption process by reducing adsorption sites interaction through the binding of Na+ and Cl- ions to the zwitterionic form of TC [29].

The adsorption efficiency of antibiotics by SA/CMCS hydrogel microbeads increased with pH due to the higher deprotonation rate of -COOH to -COO^−^ under alkaline conditions [18], which effectively promotes the binding of positively charged antibiotic molecules through electrostatic interactions. Conversely, at lower pH (acidic conditions), the protonation of -NH_2_ and -NH^+^ groups in the SA/CMCS molecules reduces the negative charge of the hydrogel microbeads, further decreasing the adsorption of cationic antibiotics. These findings align with the results from the zeta potential analysis in Figure 1b.

In binary antibiotic systems, the adsorption of TC and CIP becomes more complex due to competitive adsorption for binding sites. As shown in Figure 8b, the adsorption data for SC-400 in 50 mL of 100 mg/L TC and CIP compound solutions demonstrate that the adsorption of TC gradually decreases in the binary solution due to competitive adsorption with CIP. TC has more phenolic functional groups, while CIP has more ketonic functional groups, making it less comparable to CIP [30]. With the increase in SC-400, the adsorption of CIP decreases significantly, indicating that TC and CIP compete for the limited binding sites and the competition becomes more pronounced with the increase in SC-400.

Compared to single antibiotic solutions, the presence of CIP reduces TC adsorption by competing for binding sites on SC-400. Additionally, with the increase in SC-400, the adsorption capacity of CIP in the binary system slightly decreases compared to the single system. From a time perspective, CIP exhibits a higher adsorption efficiency than TC and occupies binding sites more quickly due to its faster adsorption rate. Thus, when CIP and TC coexist in a solution, the adsorption capacity of CIP is almost unaffected by the presence of TC. In contrast, the adsorption capacity of TC decreases more significantly than that of CIP, indicating that the primary competitive effect is related to the individual compound’s affinity for the adsorbent.

## 3. Conclusions

In this work, SA/CMCS hydrogel microbeads were successfully fabricated using the electrostatic spray (ES) technique. The adsorption of antibiotics TC, and CIP by the hydrogel microbeads was studied by understanding the effect of pH and temperature. A suitable adsorption condition at pH 7 and 25 °C provides maximum adsorption capacity and further increase of temperature weakens the electrostatic interaction between the hydrogel microbeads and antibiotics. In a single antibiotic solution, the pseudo-second order model provides better accuracy in describing the adsorption behavior of both TC and CIP. The study observed that the adsorption process occurred in physical interaction, followed by relatively straightforward interaction. Furthermore, the intraparticle diffusion model showed that the TC antibiotics are more complex and involve multiple adsorption and desorption processes. For the adsorption isotherm, both single and binary systems expressed that the Freundlich and Temkin isotherm models give the highest accuracy and R^2^. The analysis of hydrogel microbeads under FTIR shows some shifted bonds of C-C double bond and C-O bond, thus expressing the adsorption process on the microbeads. TC has lower adsorption due to highly competition with CIP in the binary system. A detailed analysis of the microbeads structure effect with high size variation is suggested to provides a clearer understanding of antibiotics adsorption. Smaller size of hydrogel microbeads (SC-400) significantly enhances adsorption capacity in both single and binary antibiotic systems due to the increase in surface area and interaction sites. This study revealed that SA/CMCS hydrogel microbeads have good potential for antibiotic adsorption in various applications, including drug delivery, protein binding, and wastewater treatment, based on high adsorption rate, especially for a smaller size of microbeads.

## 4. Materials and Methods

### 4.1. Material

Sodium alginate (SA) and carboxymethyl chitosan (CMCS) were purchased from Macklin (Shanghai, China). The antibiotics of tetracycline and ciprofloxacin, hydrochloric acid (HCl), sodium hydroxide (NaOH), and sodium chloride (NaCl) were purchased from Aladdin (Shanghai, China). The preparation and synthesis of hydrogel microbeads were performed using a dehumidifying electrostatic spraying machine SNXJ-1400 equipped with 20G needles from Spinning Intelligent Equipment (Qingdao, China).

### 4.2. Preparation of the SA/CMCS Microbeads Using Electrostatic Spraying Method

The preparation of hydrogel microbeads was conducted by following the previous method [31]. SA and CMCS were each dissolved in deionized water at a 1:1 weight ratio, with final concentration of 1.5 wt% for both polymers. The mixture of SA/CMCS was then electrostatically sprayed using a SNXJ-1400 dehumidifying electrostatic spraying machine, equipped with 20G needles and operated at voltages of 0 and 13.0 kV. A voltage of 0.0 kV resulted in larger beads (SC-2000; ~2000 µm), whereas 13.0 kV produced smaller beads (SC-400 ~400 µm). A voltage of 13.0 kV was selected as optimal for obtaining uniform, small-diameter microbeads suitable for adsorption studies, as shown in Figure 5. An acidic bath at 0.1 N HCl solution was placed beneath the needles to facilitate the formation of SA/CMCS hydrogel microbeads. The hydrogel microbeads were allowed to solidify in 0.1 N HCl solution for 4 h and rinsed with deionized water until reaching pH 7.0. The prepared hydrogel microbeads were freeze-dried and stored in desiccator at room temperature to avoid moisture absorption before further characterization and antibiotic adsorption study. In this study, the voltage was adjusted to 0.0 and 13.0 kV for the synthesis of SA/CMCS microbeads.

### 4.3. Adsorption Study

The antibiotic adsorption experiments were conducted in 50 mL single or binary antibiotic solutions under constant conditions of 25 °C and 80 rpm. The initial concentrations of tetracycline (TC) and ciprofloxacin (CIP) in single or binary solutions were set to 20 mg·L^−1^. The binary solution was set at equal concentration in a volume ratio of 1:1. To determine the optimal conditions for antibiotic adsorption, two factors, pH (3–9) and temperature (15, 25, and 35 °C), were evaluated for their effects on adsorption efficiency. For the effect of pH on antibiotic adsorption, 50 mL of 20 mg·L^−1^ single or binary antibiotic solutions were adjusted to a pH range of 3 to 9 using 0.1 M HCl and NaOH, while the temperature was kept constant at 25 °C. Subsequently, 10 mg of electrostatically sprayed SA/CMCS hydrogel microbeads (SC-2000 and SC-400) were added to the solution. To investigate the effect of temperature on antibiotic adsorption, 50 mL of 20 mg·L^−1^ single or binary antibiotic solutions were treated with 10 mg of electrostatically sprayed SA/CMCS hydrogel microbeads at 15, 25, and 35 °C, while maintaining a pH of 7. The subsequent experimental steps were similar to those described above, and the concentrations were analyzed using UV-Vis and LC-MS for single and binary solutions. The thermodynamic properties of antibiotic adsorption were analyzed using the Gibbs–Helmholtz equation as follows:

(1) $$∆G=-\mathrm{RT}\;\ln K_{L}$$

(2) $$K_{L}=\frac{q_{e}}{C_{e}}$$

The thermodynamic parameter, Δ*G*, refers to the standard Gibbs energy change, *R* is the universal gas constant, *T* is the temperature, *K*_L_ is the Langmuir isotherm constants, *q*_e_ is the amount adsorbed at the equilibrium (mg·g^−1^), and *C*_e_ is the equilibrium concentration in solution (mg·L^−1^). Meanwhile, the enthalpy, ∆*H*, and entropy, ∆*S*, were obtained from the following equation:

(3) ∆G=∆H−T∆S

In the adsorption kinetics study, the mixtures were placed on a constant-temperature shaker (25 °C), and supernatant samples were taken at 10, 20, 30, 40, 50, 70, 100, 150, and 270 min by filtration using a 0.22 μm syringe filter. Pseudo-first order and pseudo-second order kinetic models shown in Equations (4) and (5) were used to fit the adsorption of single and binary systems. *q*_e_ and *q*_t_ are the amount of adsorbate adsorbed at equilibrium and at any given time, *t*, while *k*_1_ and *k*_2_ refer to the rate constant.

(4) $$\text{Pseudo-first-order}:\;\ln\left(q_{e}\;-\;q_{t}\right)=\ln q_{e}-k_{1}t$$

(5) $$\text{Pseudo-second-order}:\;\frac{t}{q_{t}}=\frac{1}{k_{2}q_{e}^{2}}+\frac{t}{q_{e}}$$

Intraparticle diffusion model was conducted to increase the understanding in the adsorption behavior of antibiotics and determine the role of diffusion in the adsorption, as mentioned in Equation (6), where *q*_t_ is the amount of adsorbate adsorbed at any given time, *t*, *k* represents the intraparticle diffusion rate (mg/g/min), and *C* is a constant.

(6) $$q_{t}=kt^{0.5}+C$$

In the adsorption isotherm study, single antibiotic solution was prepared with initial concentrations of 10, 20, 40, 60, 100, 150, and 200 mg·L^−1^, and binary antibiotic solutions were prepared with TC and CIP coexisting at respective concentrations of 10, 20, 40, 60, 100, 150, and 200 mg·L^−1^ with the volume ratio of 1:1 and equal concentration. From these solutions, 50 mL of single or binary antibiotic solutions was taken, adjusted to pH 7 based on the maximum adsorption observed in preliminary studies, and mixed with 10 mg of electrostatically sprayed SA/CMCS hydrogel microbeads. The mixtures were placed in a constant-temperature shaker at 25 °C with a rotation speed of 80 rpm for 270 min. The adsorption isotherm study was fitted in Equations (7)–(9) as follows:

(7) $$\mathrm{Langmuir}\;\mathrm{model}:\;\frac{C_{e}}{q_{e}}=\frac{1}{K_{L}q_{m}}+\frac{C_{e}}{q_{m}}$$

(8) $$\mathrm{Freundlich}\;\mathrm{model}:\;\ln q_{e}=\ln K_{F}+\frac{1}{n}\ln C_{e}$$

(9) $$\mathrm{Temkin}\;\mathrm{model}:\;q_{e}=\frac{\mathrm{TR}}{b}\ln K_{T}+\frac{\mathrm{TR}}{b}\ln C_{e}$$

where *q*_m_ is the theoretical maximum MB adsorption capacity (mg·g^−1^), *K*_L_ is the Langmuir adsorption constant (L/mg), *C*_e_ is the MB concentration at equilibrium (mg·L^−1^), *K*_F_ and *n* are the Freundlich constants, *R* is the ideal gas constant (8.314 J/mol·K), *K*_T_ is the Temkin constant, and *b* is the Temkin constant related to the heat of adsorption.

All supernatant samples were filtered through syringe filters (organic, 0.22 μm), and the antibiotic concentrations of TC and CIP in single solutions were measured using UV-Vis spectrophotometer (Thermo Scientific, Waltham, MA, USA, GENESYS 10S) at 275 nm (TC) and 350 nm (CIP). For binary antibiotic solutions, detection was performed using high performance liquid chromatography (HPLC). The adsorption solution was filtered through a 0.22 μm organic membrane before being injected into the HPLC system. An aliquot of 10 μL of the filtered sample was injected into a ZORBAX SB-C18 (Agilent, Santa Clara, CA, USA) column. The mobile phase consisted of Solvent A (0.01 mol/L potassium dihydrogen phosphate solution), Solvent B (methanol), and Solvent C (pure water), delivered at a flow rate of 0.8 mL/min with a column temperature maintained at 20 °C. The detection wavelengths were set at 350 nm for CIP and 275 nm for TC. The gradient elution program was performed as follows: the mobile phase consisted of 80% Solvent A and 20% Solvent B for 10 min; 90% Solvent C was introduced for 10 min; 100% Solvent B was maintained for another 10 min; and the elution returned to the initial composition of 80% Solvent A and 20% Solvent B for the last 10 min.

### 4.4. Characterization of Sorbents

The size of microbeads was measured using image analysis software. The image was captured and being analyzed using ImageJ (Version 2.15.1) using open-source Fiji software (https://imagej.net/software/fiji/downloads, accessed on 4 July 2025) to measure the size of single microbeads. The chemical structures of the electrostatically sprayed microbeads were identified using Fourier-transform infrared (FTIR) spectroscopy (Thermo Scientific, Waltham, MA, USA, Nicolet iS10) in a wavelength range of 400–4000 cm^−1^, resolution of 2 cm^−1^, and 32 scans. The zeta potential of the hydrogel microbeads was measured using a Zetasizer Nano ZS (Malvern Instruments, Malvern, UK) to determine their surface charge properties. To identify the effect of ionic strength on the adsorption capacity of hydrogel microbeads, NaCl solutions at different concentrations (0.01 M, 0.03 M, and 0.05 M) were added to a 100 mg/L antibiotic solution. Subsequently, 10 mg of SC-400 microbeads was introduced into 50 mL of the NaCl-containing antibiotic solution, and the mixture was stirred at 25 °C with a rotation speed of 80 rpm. After reaching saturation, the solution was filtered, and the residual antibiotic concentration was measured. The mutual interference between the two antibiotics of single and binary was determined by varying microbeads masses (5, 10, 15, 20, 25, 30, 35, and 40 mg) and added to a 100 mg/L antibiotic solution. The microbeads were placed into 50 mL of the antibiotic solution and stirred at 25 °C with a rotation speed of 80 rpm. After reaching saturation, the solution was filtered, and the residual antibiotic concentration was measured.

## Figures and Tables

**Table 1:** Thermodynamic results for single and binary adsorption systems.

Systems	Temperature, °C	Adsorption Equilibrium Constant, K_L_ (L·mg)	Gibbs Free Energy ∆G (kJ·mol^−1^)	Enthalpy, ∆H (kJ·mol^−1^)	Entropy ∆S (J·mol^−1^K^−1^)
Single TC	15	1.2998 ± 0.1040	−0.628 ± 0.192	46.213	152.5
	25	1.1611 ± 0.0929	−0.370 ± 0.198		
	35	1.0665 ± 0.0853	−0.165 ± 0.205		
Single CIP	15	20.8865 ± 1.0443	−7.281 ± 0.120	43.462	177.2
	25	55.0970 ± 2.7549	−9.938 ± 0.124		
	35	68.4252 ± 3.4213	−10.826 ± 0.128		
Binary TC	15	0.8700 ± 0.0870	0.334 ± 0.240	8.769	45.5
	25	0.7598 ± 0.0760	0.681 ± 0.248		
	35	0.8475 ± 0.0848	0.424 ± 0.256		
Binary CIP	15	13.7001 ± 0.4110	−6.270 ± 0.072	4.342	20.6
	25	15.3870 ± 0.4616	−6.776 ± 0.074		
	35	13.5764 ± 0.4073	−6.682 ± 0.077		

**Table 2:** Fitted parameters for adsorption kinetic models in single and binary systems.

System	Kinetics	Parameter	SC-2000-TC	SC-400-TC	SC-2000-CIP	SC-400-CIP
Single	*q*_e_ experimental (mg·g^−1^)		257.0453 ± 10.72	273.7004 ± 9.04	343.9326 ± 11.15	373.9326 ± 10.85
	Pseudo-first order	*q*_e_ (mg·g^−1^)	255.9641 ± 1.08	262.9692 ± 10.73	361.6163 ± 17.68	388.0916 ± 14.15
		*k*_1_ (min^−1^)	0.0314 ± 0.003	0.0480 ± 0.005	0.0415 ± 0.004	0.0573 ± 0.006
		*R* ^2^	0.8334	0.9483	0.9327	0.9130
	Pseudo-second order	*q*_e_ (mg·g^−1^)	305.1926 ± 48.14	297.4490 ± 23.74	413.3961 ± 69.46	432.6443 ± 58.71
		*k*_1_ (min^−1^)	0.000113	0.000218	0.000128	0.000187
		R^2^	0.7663	0.9461	0.8217	0.7725
	Intraparticle diffusion	*k*_a_ (mgg^−1^min^−0.5^)	123.2359 ± 12.32	72.8009 ± 7.28	74.5345 ± 7.45	104.6058 ± 10.46
		*R*_a_^2^	1.0000	1.0000	0.9870	0.9970
		*k*_b_ (mgg^−1^min^−0.5^)	37.7781 ± 3.77	13.4903 ± 1.34	0.0601 ± 0.01	1.1654 ± 0.11
		*R*_b_^2^	0.8756	0.9838	0.7666	0.8176
		*k*_c_ (mgg^−1^min^−0.5^)	2.5993 ± 0.25	2.5347 ± 0.25		
		*R*_c_^2^	0.9372	0.9828		
Binary	*q*_e_ experimental (mg·g^−1^)		239.1359 ± 7.47	273.7004 ± 13.11	350.2247 ± 14.16	376.6292 ± 10.37
	Pseudo-first order	*q*_e_ (mg·g^−1^)	255.9641 ± 16.82	262.9692 ± 10.73	361.6163 ± 11.39	390.4361 ± 13.80
		*k*_1_ (min^−1^)	0.0314 ± 0.003	0.0480 ± 0.004	0.0415 ± 0.004	0.0693 ± 0.006
		*R* ^2^	0.7663	0.9461	0.8217	0.7725
	Pseudo-second order	*q*_e_ (mg·g^−1^)	305.1926 ± 66.05	297.4490 ± 23.74	413.3961 ± 63.17	432.6443 ± 56.01
		*k*_1_ (min^−1^)	0.000113	0.000218	0.000128	0.000187
		*R* ^2^	0.80958	0.94088	0.92309	0.74625
	Intraparticle diffusion	*k*_a_ (mgg^−1^min^−0.5^)	59.2387 ± 5.92	93.8703 ± 9.38	127.2468 ± 12.72	200.8108 ± 20.08
		*R*_a_^2^	0.9186	0.7540	0.9965	1.0000
		*k*_b_ (mgg^−1^min^−0.5^)	17.6819 ± 1.76	68.3053 ± 6.83	−0.1538 ± −0.01	1.3127 ± 0.13
		*R*_b_^2^	0.8179	0.7792	0.7792	0.7434
		*k*_c_ (mgg^−1^min^−0.5^)	−0.1598 ± 0.015	−1.1353 ± 0.11		
		*R*_c_^2^	0.5686	0.9432		

**Table 3:** Fitted parameters of adsorption isotherm model for different microbeads size in single and binary systems.

Systems	Isotherms	Parameters	SC-2000-TC	SC-400-TC	SC-2000-CIP	SC-400-CIP
Single	Langmuir	*q_m_* (mg·g^−1^)	200.3854 ± 10.0193	289.6927 ± 8.6908	399.4930 ± 19.9727	436.0794 ± 21.8040
		*K_L_* (L·g^−1^)	0.0286 ± 0.0014	0.9821 ± 0.0295	0.8957 ± 0.0448	0.8569 ± 0.0429
		*R* ^2^	0.8835	0.9378	0.8605	0.8517
	Freundlich	*K_F_* (mg^1−N^·L^N^·g^−1^)	21.3941 ± 0.2139	40.5521 ± 0.4055	206.5262 ± 10.3263	207.8708 ± 10.3935
		*n*	−0.4609 ± 0.0046	−0.3839 ± 0.0038	−0.1531 ± 0.0077	−0.1697 ± 0.0085
		*R* ^2^	0.9851	0.9801	0.8826	0.8435
	Temkin	*b* (J·mol^−1^)	0.2678 ± 0.0134	0.1654 ± 0.0050	81.6424 ± 4.0821	12.1640 ±0.6082
		$K_{T}$ (L·g^−1^)	55.2808 ± 2.7640	88.3751 ± 2.513	45.7654 ± 2.2883	66.8990 ± 3.3450
		R^2^	0.8437	0.9035	0.8924	0.8740
Binary	Langmuir	*q_m_* (mg·g^−1^)	241.2161 ± 12.0608	289.6927 ± 8.6908	350.0511 ± 17.5026	436.0769 ± 21.8039
		*K_L_* (L·g^−1^)	0.0256 ± 0.0013	0.0180 ± 0.0005	1.0318 ± 0.0516	0.1545 ± 0.0077
		*R* ^2^	0.8472	0.9378	0.8495	0.8552
	Freundlich	*K_F_* (mg^1−N^·L^N^·g^−1^)	20.7733 ± 1.0387	14.1002 ± 0.7050	179.5854 ± 5.3875	207.8769 ± 10.3939
		*n*	−0.5047 ± 1.0387	−0.6093 ± 0.0305	−0.1810 ± 0.0054	−0.1697 ± 0.0085
		*R* ^2^	0.8349	0.8608	0.9184	0.8435
	Temkin	*b* (J·mol^−1^)	0.2154 ± 0.0065	0.1579 ± 0.0047	215.5266 ±6.4658	23.4658 ± 1.1733
		$K_{T}$ (L·g^−1^)	70.3573 ± 2.1107	84.5191 ± 2.5356	38.7629 ± 1.1629	58.5195 ± 2.9260
		*R* ^2^	0.9156	0.9057	0.9414	0.8718

## Data Availability

The original contributions presented in this study are included in the article. Further inquiries can be directed to the corresponding authors.

## Abbreviations

The following abbreviations are used in this manuscript:

SA	Sodium alginate
CMC	Carboxymethyl cellulose
TC	Tetracycline
CIP	Ciprofloxacin
